# Electrochemical Dehydration Reaction

**DOI:** 10.1002/cssc.202501552

**Published:** 2025-09-10

**Authors:** Johannes Schneider, Enrico Lunghi, Siegfried R. Waldvogel

**Affiliations:** ^1^ Department of Chemistry Johannes Gutenberg University (JGU) Duesbergweg 10–14 55128 Mainz Germany; ^2^ Department of Electrosynthesis Max‐Planck‐Institute for Chemical Energy Conversion Stiftstraße 34–36 45470 Mülheim an der Ruhr Germany; ^3^ Institute of Biological and Chemical Systems – Functional Molecular Systems (IBCS–FMS) Karlsruhe Institute of Technology (KIT) Kaiserstraße 12 76131 Karlsruhe Germany

**Keywords:** carboxylic acids, dehydrative reactions, electrolysis, sulfonic acids, sustainable chemistry

## Abstract

Electrochemical dehydration reaction is a fascinating and underexplored field of research, which has started to attract significant attention in recent years. Dehydration reactions are characterized by the formal removal of water in the course of the transformation, and they are among the most fundamental types of reactions found throughout chemistry. Examples are esterification reactions, amidation reactions, and the synthesis of carbon‐heteroatom multiple bonds. In general, dehydration reactions are not considered to be redox reactions, because no oxidation states change in the substrate from which water is eliminated or in the dehydration reagent that is utilized. At first glance, there does not seem to be a link between dehydration reactions and redox chemistry. In recent years, however, it has been demonstrated that dehydration reactions can be carried out by electrolysis. Given the enormous importance of dehydration reactions from academic to technical scale, electrochemical dehydration reactions offer a more sustainable approach to such transformations. In this review, the recent progress is surveyed and the opportunities of this new and evolving field are highlighted. Electrochemical dehydration reactions are an interesting new discipline in the emerging domain of electroorganic chemistry, which is currently experiencing a remarkable renaissance to establish itself as a 21st‐century technique.

## Graphical abstract

Electrochemical dehydration reactions are a fascinating and underexplored field of research in the domain of electrosynthesis. They offer a sustainable alternative to hazardous and harsh dehydration reagents. In this review, the recent progress that has been made in this emerging research topic is surveyed.

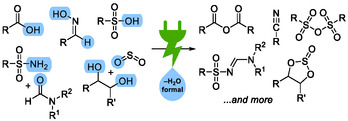

## 1 Introduction

**Scheme 1:**
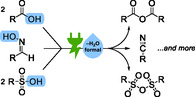
Some examples of electrochemical dehydration reactions.

Dehydration reactions are conventionally performed using an excess of a dehydration reagent, which results in harsh reaction conditions and in the generation of hazardous reagent waste.^[^ ^1^ ^]^ This also complicates the downstream processing.^[^ ^2^ ^]^ Electrosynthesis is a first‐class technique for replacing such dangerous and waste intense transformations with safer electrochemical alternatives which are characterized by milder reaction conditions.^[^ ^3^, ^4^ ^]^ One of the many advantages of electrosynthesis is the direct use of electricity as a comparably inexpensive and universal redox agent, which avoids the generation of reagent waste utilizing anodic oxidation and cathodic reduction reactions instead of oxidizing or reducing agents.^[^ ^5^ ^]^ Since the chemical bonds consist of electrons, the addition of an electron to a bond (reduction) or the removal of an electron from a bond (oxidation) activates the substrates for chemical conversion. This can proceed either via Brønsted acid/base or by radical‐type reactivity.^[^ ^6^ ^]^ The avoidance of metal catalysts^[^ ^7^ ^]^ and the decreased number of synthesis steps make electrosynthesis an innovative production technology which goes way beyond other sustainable improvements.^[^ ^8^ ^]^ Furthermore, the transformation usually occurs at the electrodes or close by them. Switching off the electricity stops anodic oxidation and cathodic reduction and helps to prevent runaway reactions, which adds an inherent safety aspect to electrosynthetic transformations.^[^ ^9^ ^]^ In addition, galvanostatic electroorganic transformations are typically simple to scale‐up.^[^ ^10^ ^]^ In the field of electrosynthesis, dehydrative transformations have not been in the focus of research until recently. Most likely, such reactions are more common than currently known, and they might be facilitated by the strong electrical field close to the electrodes. In this regime, the nature of the electrolyte plays an utmost important role.^[^ ^11^ ^]^ Especially in the context of green chemistry, dehydration reactions have often been discussed as chemical processes for which significantly more innovation is required.^[^ ^12^ ^]^ One of the major advantages of dehydration reactions is that water is one of the most uncritical and simple leaving groups, because disposal of water is much easier than the disposal of reagent waste originating from other leaving groups such as mesylates, triflates, or tosylates.^[^ ^13^ ^]^ In addition, water is not toxic, corrosive, or environmentally harmful. As in any dehydration reaction, the activation of the OH group in the substrate to form a good leaving group is the key. While many conventional dehydration reactions employ an excess of dehydration agent, it would be advantageous to generate the dehydration reagent in situ by electrolysis. In other electrochemical dehydration reactions, the electrolysis acts as an activation step for the substrate to trigger a subsequent dehydration reaction, in which case no electrochemically generated dehydration reagent is involved. The electrogeneration of reagents is already a well‐established concept in other areas of electrosynthesis,^[^ ^14^ ^]^ for example, for the generation of carbocations^[^ ^15^ ^]^ or bases.^[^ ^16^ ^]^ The overall simplicity of electrochemical dehydration reactions can also contribute to their atom economy.^[^ ^17^ ^]^ The basic underlying concept of electrochemical dehydration reactions offers fascinating opportunities for other new applications: electrolysis is used as the key technique to apply the thermodynamic driving force to carry out transformations which are typically not associated with redox reactions. Electrochemical dehydration reactions should therefore be viewed as a source of inspiration for other types of reactions that are not redox reactions but still might be carried out via electrolysis. This will lead to applications of electrosynthesis in areas where electrolysis was not considered as the key technology before.^[^ ^18^ ^]^ In this review, we discuss notable examples of electrochemical dehydration reactions, and we propose how to classify them mechanistically (**Scheme** **1**).

## 2 Electrochemical Dehydration of Carboxylic Acids to their Anhydrides

**Scheme 2:**
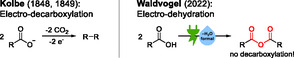
Comparison of Kolbe electrolysis (left) with electrochemical dehydration of carboxylic acids to their carboxylic anhydrides (right).^[^ ^19^, ^28^ ^]^

In 2022, our group reported a conceptually new and nondecarboxylative electrochemical synthesis of carboxylic anhydrides from carboxylic acids (**Scheme** **2**).^[^ ^19^ ^]^ This result was rather surprising, because the electrolysis of carboxylic acids, known as the Kolbe electrolysis, has been known and studied for more than 170 years at the time of our discovery. The Kolbe electrolysis is one of the very few named reactions from the field of organic electrosynthesis that is known to even those organic chemists who are not working with electrolysis.^[^ ^20^ ^]^ Even more so, Hermann Kolbe's pioneering publications from 1848 and 1849 mark a cornerstone of organic electrochemistry,^[^ ^21^ ^]^ and the electrolysis of carboxylic acids has since then been a major research topic.^[^ ^22^, ^23^ ^]^ Nonetheless, a dehydrative reaction pathway when electrolyzing carboxylic acids that is not accompanied by a decarboxylation step has not been reported before 2022.^[^ ^19^ ^]^

**Scheme 3:**
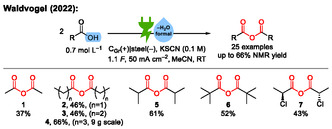
Examples from the electrochemical dehydration of carboxylic acids to their anhydrides. RT = room temperature.^[^ ^19^ ^]^

Importantly, the Kolbe reaction is always characterized by a decarboxylation step, which means that one carbon atom of the carboxylic acid function in the starting material is eliminated as CO_2_ in the course of the reaction.^[^ ^24^ ^]^ In contrast, the electrochemical synthesis of carboxylic anhydrides from their carboxylic acids that was found by our group is a nondecarboxylative dehydration reaction, and all carbon atoms of the starting material are preserved in the product anhydride (**Scheme** **3**). Noteworthy, for the Kolbe electrolysis, at least a partial neutralization of the acid is necessary.^[^ ^24^ ^]^ However, in our approach, the electrolysis is carried out without base, and only a supporting electrolyte is added. No conventional dehydration reagent is utilized, and the hydrogen evolution reaction (HER) at the cathode could be detected. The electrochemical valorization of carboxylic acids is interesting because they are naturally abundant, nontoxic, readily available, and inexpensive starting materials.^[^ ^22^ ^]^ In general, the carboxylic anhydrides which are obtained by electrolysis are highly valuable reagents for organic synthesis, and they are commonly utilized, for example, in acylation reactions of amines for the synthesis of amides. Such amidation reactions are of utmost importance from academic to technical scale,^[^ ^25^ ^]^ especially in the context of medicinal chemistry.^[^ ^26^ ^]^

In the initial publication, 25 carboxylic anhydrides were reported with NMR yields (obtained by ^1^H nuclear magnetic resonance spectroscopy) up to 66%.^[^ ^19^ ^]^ Notably, the selectivity of this electrochemical synthesis of carboxylic anhydrides from carboxylic acids can be very high, in some cases over 95%. A graphite (C_Gr_) anode and a stainless steel cathode are used as inexpensive and environmentally friendly electrode materials.^[^ ^27^ ^]^ The electrolysis is carried out under mild reaction conditions, because neither heating nor cooling increases the yield of anhydride, and no other additive than a thiocyanate supporting electrolyte is required. The reaction conditions tolerate double bonds and halo substituents in the substrate. Notably, the electrolysis requires only a small amount of applied charge (1.1 *F*) in combination with a high current density of 50 mA cm^−2^. Such conditions allow for fast electrolysis. The mechanistic concourse at that time was not identified yet, and in particular, the fate of the oxygen in the carboxylic acid starting material was unknown. No intermediates or side products which would give any hint about the reaction mechanism besides hydrogen could be detected.^[^ ^28^ ^]^ In particular, typical products of a Kolbe electrolysis or a Hofer–Moest pathway were absent. Since this reaction is a dehydration reaction, the key to understanding the mechanism is to analyze where the water remains that is eliminated during the electrolysis of the carboxylic acid to form the carboxylic anhydride. This is an interesting question because the reaction is carried out only with the addition of a supporting electrolyte, and no conventional dehydration reagent or other reagents are added. Acetonitrile is used as a solvent, but it does not act as a dehydration reagent, because the reaction works as well in other solvents such as acetone. By control experiments, it could be ruled out that oxygen is evolving from the solution during electrolysis. At the cathode, the HER is observed, which is quite common.^[^ ^29^ ^]^ Thiocyanate‐supporting electrolytes give the highest yields of anhydrides, although other supporting electrolytes also result in anhydride formation, albeit with lower yields. Several electrode materials can be chosen, and the reaction is not limited to a specific combination of materials.^[^ ^27^ ^]^ Cyclic voltammetry measurements revealed the irreversible oxidation of the thiocyanate‐supporting electrolyte. When performing the reaction in the anodic compartment of a divided cell instead of an undivided cell setup, only traces of anhydride are found. In general, the reaction conditions are mild and simple, because the electrolysis is carried out at room temperature (RT) under air. It could be demonstrated in control experiments that the carboxylic anhydride product decomposes again to the carboxylic acid upon electrolysis. Interestingly, a yield higher than 50% could be achieved when using less than 0.5 equivalents of thiocyanate‐supporting electrolyte, which indicates that if thiocyanate was responsible for the electrochemical dehydration, one equivalent of thiocyanate can react with more than one equivalent of carboxylic acid in the course of the electrolysis to form the carboxylic anhydride. Most importantly, the reaction does not work at all without electricity.

**Scheme 4:**
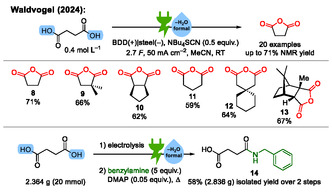
Examples from the electrochemical dehydration of dicarboxylic acids to their cyclic anhydrides.^[^ ^28^ ^]^ BDD = boron‐doped diamond. DMAP = 4‐(dimethylamino)pyridine.^[^ ^59^ ^]^

**Scheme 5:**
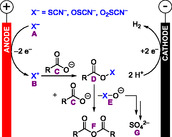
Proposed mechanism for the electrochemical dehydration of carboxylic acids to their anhydrides.^[^ ^28^ ^]^ By ^18^O isotope labeling of the carboxylic acid starting material, ^18^O‐labeled sulfate was detected in the reaction solution after electrolysis.

Subsequently, this research was extended to dicarboxylic acids, which are electrochemically dehydrated to the corresponding cyclic anhydrides (**Scheme** **4**).^[^ ^28^ ^]^ In addition, we carried out mechanistic investigations which allowed us to propose a mechanism (**Scheme** **5**). In total, 20 examples were demonstrated with NMR yields up to 71%. The cyclic carboxylic anhydrides which were generated in situ by electrolysis were employed in amidation reactions by the addition of amines. The products of such acylation reactions are amides with a ω‐carboxylic acid side chain, which allows for further functionalization. Such addition reactions of amines to cyclic anhydrides offer a favorable atom economy, because no reagent waste from the cyclic carboxylic anhydride is generated.^[^ ^17^ ^]^

In these mechanistic investigations, we found that the electrochemical dehydration proceeds via anodic oxidation of the thiocyanate‐supporting electrolyte **A** to **B**, which acts as an electrogenerated dehydration reagent (Scheme 5).^[^ ^28^ ^]^ **B** then reacts with one equivalent of carboxylate **C** to an active ester **D**. Another equivalent of **C** reacts with **D** to form the carboxylic anhydride **F**, while **E** is eliminated as a good leaving group. Most importantly, one equivalent of thiocyanate can take up several equivalents of water in the course of the electrochemical dehydration reaction, which is accompanied by the formation of increasingly oxygenated sulfur species **E**. In the last step, sulfate **G** is formed. The sulfate is unequivocally detected by high‐resolution mass spectrometry with ^18^O isotope‐labeled dicarboxylic acid as starting material, since ^18^O‐labeled sulfate was found.^[^ ^28^ ^]^ This proposed mechanism represents a simplified picture, because many of the oxygenated sulfur species **E** are known to undergo a variety of other reactions.^[^ ^30^ ^]^

## 3 Electrochemical Catalytic Dehydration of Carboxylic Acids to their Anhydrides

**Scheme 6:**
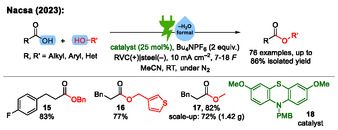
Examples from the electrochemical esterification of carboxylic acids with alcohols to esters.^[^ ^31^, ^32^ ^]^ PMB = *p*‐methoxy benzene. The scale‐up was carried out via electrolysis under constant cell voltage.

The group of Nacsa reported an electrochemical design for catalytic dehydration of carboxylic acids with alcohols to obtain esters in 2023 (**Scheme** **6**).^[^ ^31^, ^32^ ^]^ This interesting report came after our findings of the electrochemical dehydration of carboxylic acids in 2022,^[^ ^19^ ^]^ and it uses a different approach. Instead of thiocyanates in a dual role as supporting electrolyte and electrochemically generated dehydration reagent,^[^ ^11^ ^]^ the group of Nacsa utilized an electron‐rich phenothiazine derivative **18** as catalyst for electrochemically driven catalytic dehydration of carboxylic acids with alcohols to the corresponding esters. Notably, both our approach and the approach by Nacsa are realized based on sulfur compounds. Nacsa attempted to utilize catalysts based on phosphorous, but these attempts were unsuccessful because of the difficult reduction of the P=O bond.^[^ ^33^ ^]^ No acid or base is required as an additive, and the electrolysis is carried out at RT using a catalyst loading of 25 mol%. Reticulated vitreous carbon is used as anode material and stainless steel as cathode material. The highest yields are obtained when nitrogen is utilized as inert gas. In total, 76 examples are demonstrated with isolated yields up to 86%.

**Scheme 7:**
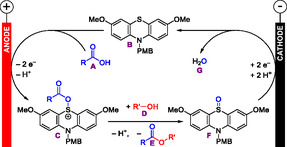
Proposed mechanism of the phenothiazine‐mediated electrochemical catalytic esterification of carboxylic acids with alcohols to esters.^[^ ^31^, ^32^, ^34^ ^]^ PMB = *p*‐methoxy benzene.

Nacsa noted a comparably high amount of applied charge that is required (7 to 18 *F*), which is attributed to electron transfer between the electrodes by the phenothiazine catalyst **18**, which does not contribute to the conversion of substrates. Such a large amount of required charge prolongs electrolysis. The scalability of the transformation was demonstrated, and an analysis of stereochemical outcome demonstrated the retention of configuration. As depicted in **Scheme** **7**, Nacsa proposed that the phenothiazine‐based catalyst **B** undergoes anodic oxidation to react with the carboxylic acid **A** to a sulfonium species **C**, which activates the OH group of the carboxylic acid so that nucleophilic acyl substitution with an alcohol **D** can take place. The product of this step is an ester **E** and the corresponding sulfoxide (phenothiazine‐*S*‐oxide) **F**. Cathodic reduction of sulfoxide **F** regenerates the catalyst **B** by elimination of water **G**, which is the driving force for catalytic turnover. It should be mentioned that further oxidation of the sulfoxide **F** to the corresponding sulfone is possible in theory as well. Such a separate catalytic cycle based on the sulfoxide **F** and sulfone could also contribute to conversion of the substrate.

**Scheme 8:**
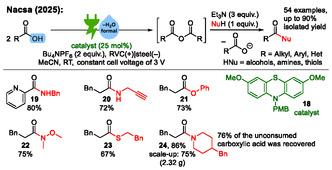
Examples from the electrochemical dehydration of carboxylic acids to carboxylic anhydrides for the synthesis of amides, esters, and thioesters.^[^ ^34^ ^]^

Nacsa modified this approach in 2025 into a carboxylic acid substitution platform for the synthesis of amides, esters, and thioesters utilizing carboxylic anhydrides as key intermediates (**Scheme** **8**).^[^ ^34^ ^]^ In this follow‐up work, the carboxylic anhydride is generated in situ by electrochemical dehydration of the carboxylic acid, and then, the nucleophile is added after the electrolysis has completed in a subsequent step to access the corresponding carboxylic acid derivative in a one‐pot reaction. Because nucleophiles like thiols or amines are susceptible to anodic oxidation,^[^ ^35^ ^]^ these nucleophiles were added to the reaction solution subsequently after electrolysis. In total, 54 examples are demonstrated with isolated yields up to 90%.

The reaction mechanism is similar to Nacsa's previous publication (Scheme 7). A key difference in the follow‐up publication is the subsequent addition of the nucleophile after the electrolysis has been completed. This avoids side reactions of the nucleophile which could occur if the nucleophile was present already during electrolysis. Only the electrochemical part is depicted in Scheme 7. It should be noted that Nacsa carried out this work using an electrolysis under constant cell voltage without mentioning the amount of applied charge.^[^ ^34^ ^]^ This approach was also utilized for the scale‐up reaction in the previous work (product **17** in Scheme 6). While Nacsa's work is highly interesting, such an electrolysis type is not recommended because it is not galvanostatic, and the electric current will decrease in the course of electrolysis due to the increase in internal resistance.^[^ ^36^ ^]^ This means by specifying the cell voltage and the electrolysis time, the applied charge cannot be calculated. Such an approach is also not potentiostatic because the terminal cell voltage is not equal to the electrode potential.^[^ ^37^ ^]^ Electrosynthesis should preferably be carried out as galvanostatic electrolysis by specifying the employed electric current in mA (or the current density in units of mA cm^−2^ if the electrode surface can be approximated). The amount of applied charge should be expressed as multiples of the Faraday constant (e.g., 3 *F* = 3 ⋅ 96485 C mol^−1^).^[^ ^37^ ^]^ The advantage of expressing the amount of applied charge like this is that it is independent of the amount of mol of substrate that is used, meaning that such a definition (e.g., 3 *F*) is completely independent of the scale of the electrolysis. Note also that it is *F*, not *F* mol^−1^, because the Faraday constant *F* has units of C mol^−1^, so *F* mol^−1^ would result in units of C mol^−2^.^[^ ^18^ ^]^

## 4 Electrochemical Dehydration of Aldoximes to Nitriles

**Scheme 9:**
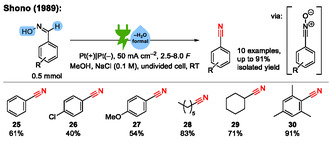
Examples from the electrochemical dehydration of aldoximes to nitriles.^[^ ^38^ ^]^

In 1989, the group of Shono published electrosynthesis of nitriles from aldoximes using halogen ions as mediators (**Scheme** **9**).^[^ ^38^ ^]^ In this report, Shono already noted the interesting link between this dehydration reaction and electrolysis: “… passing electricity is necessary … although it is formally a simple dehydration process which is unrelated to oxidation and reduction”.^[^ ^38^ ^]^ In total, ten examples are demonstrated with isolated yields up to 91%. All reactions were carried out on a 0.5 mmol scale.^[^ ^38^ ^]^

**Scheme 10:**
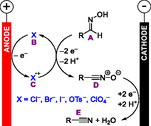
Proposed mechanism for the mediated electrochemical dehydration of aldoximes to nitriles via nitrile oxide intermediates.^[^ ^38^ ^]^

The initial reaction step is a mediated (**B**, **C**) anodic oxidation of the aldoxime **A** to a nitrile oxide **D** as intermediate, which consecutively undergoes cathodic reduction to the nitrile product **E** (**Scheme** **10**). In summary, water is eliminated from the aldoxime in the course of the electrolysis. Such a transformation is a form of consecutive paired electrolysis, and it fulfills the criteria for a domino reaction.^[^ ^39^ ^]^ Interestingly, only a limited number of such consecutive paired electrolysis are known.^[^ ^40^ ^]^ It should be noted that the term “domino reaction” was not established yet in 1989, as it was later introduced by Tietze in 1993.^[^ ^39^ ^]^ The nitrile oxide intermediates **D** are themselves valuable substrates for organic synthesis, especially for 1,3‐dipolar cycloadditions.^[^ ^41^ ^]^ Shono was unable to isolate the nitrile oxide intermediates **D**, but by utilizing a divided cell, they could be quenched by addition of styrene to form the corresponding isoxazolines in a 1,3‐dipolar cycloaddition (not shown in Scheme 10). Noteworthy, the recommended platinum electrodes corrode strongly when chloride is used as a mediator.^[^ ^42^ ^]^

**Scheme 11:**
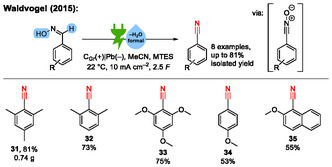
Examples from the halogen‐free electrochemical dehydration of aldoximes to nitriles. C_Gr_ = graphite. MTES = methyl triethylammonium methylsulfate.^[^ ^43^, ^44^ ^]^

Inspired by the work of Shono, in 2015, our group published an electrochemical synthesis of nitriles via a halogen‐free domino oxidation–reduction sequence.^[^ ^43^, ^44^ ^]^ In this work, the aldoxime is directly anodically oxidized without any mediator to the corresponding nitrile oxide intermediate, which is then cathodically reduced to the nitrile. In total, we reported eight substrates with isolated yields up to 81% (**Scheme** **11**).

In analogy to the work of Shono, this reaction proceeds via a domino oxidation–reduction sequence (Scheme 10), which is a form of paired electrolysis.^[^ ^3^ ^]^ In contrast to Shono's work though, no mediator is required. When using this reaction in flow electrolyzers, the use of leaded bronzes as a substitute for the soft and mechanically labile lead is recommended.^[^ ^45^ ^]^

## 5 Electrochemical Beckmann Rearrangement

**Scheme 12:**
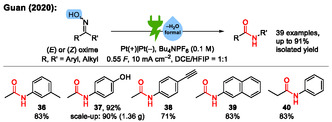
Examples from the electrochemical Beckmann rearrangement. HFIP = 1,1,1,3,3,3‐hexafluoroisopropanol, DCE = dichloroethane.^[^ ^46^ ^]^

In 2020, an electrochemical variant of the Beckmann rearrangement was reported by the Guan group (**Scheme** **12**).^[^ ^46^ ^]^ Interestingly, in this approach, the Beckmann rearrangement proceeds through a radical pathway which does not follow the *trans*‐migration rule, which states that the substituent that is trans to the oxime function in the substrate will migrate, as is the case in the classical Beckmann rearrangement. In total, 39 examples are reported with isolated yields up to 91%.

**Scheme 13:**
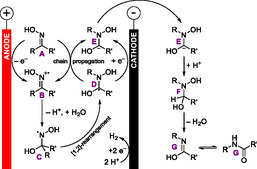
Proposed reaction mechanism of the electrochemical Beckmann rearrangement.^[^ ^46^ ^]^

The proposed reaction mechanism is depicted in **Scheme** **13**. An initial oxidation of the oxime **A** (either by anodic oxidation or by chain propagation) to **B** is followed by deprotonation and addition of water to **C**. After a radical [1,2]‐rearrangement to **D**, cathodic reduction to **E**, and protonation to **F**, water is eliminated again to form the product **G**. This is also an example of paired electrolysis. In this electrolysis, no dehydration reagent is generated.

## 6 Synthesis of *N*‐Sulfonyl Amidines by Electrochemical Dehydrative Coupling

**Scheme 14:**
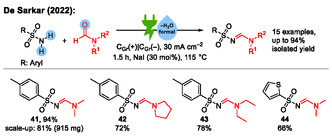
Examples from the synthesis of *N*‐sulfonyl amidines by electrochemical dehydrative coupling of primary sulfonamides with derivatives of DMF. C_Gr_ = graphite.^[^ ^47^ ^]^

In 2022, the group of De Sarkar developed an electrochemical method for the functionalization of dimethyl formamide (DMF) (**Scheme** **14**).^[^ ^47^ ^]^ In this work, electrochemically activated derivatives of DMF can react either in a dehydrative coupling reaction with a primary sulfonamide to an *N*‐sulfonyl amidine, or it can react in an oxidative cyclization reaction with hydrazides to form 1,3,4‐oxadiazoles. In this review, we focus only on the dehydrative coupling reaction to *N*‐sulfonyl amidines.

**Scheme 15:**
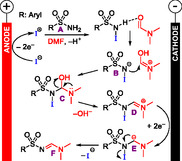
The proposed reaction mechanism of the electrochemical dehydrative coupling of primary sulfonamides with derivatives of DMF to *N*‐sulfonyl amidines.^[^ ^47^ ^]^

The electrochemical functionalization of DMF is an interesting strategy because DMF is readily available. The concept of dehydrative coupling is also interesting because in principle, water is one of the simplest and least hazardous leaving groups. The disposal of water is much simpler than the disposal of reagent waste which originates from other leaving groups such as triflates, mesylates, or tosylates.^[^ ^13^ ^]^ The authors propose an iodine‐mediated paired electrolysis as a reaction mechanism (**Scheme** **15**). Anodic oxidation of iodide leads to the formation of an iodine cation, which activates the sulfonamide **A**. By formation of an iminium ion pair **B**, water is eliminated from **C**, and the resulting iminium ion **D** is reduced again to **E**, followed by elimination of the iodide from **E**, which forms the product *N*‐sulfonyl amidine **F**.

## 7 Electrochemical Dehydration of Sulfonic Acids to their Anhydrides

**Scheme 16:**
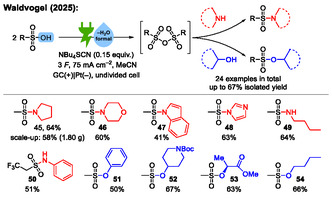
Examples from the electrochemical dehydration of sulfonic acids to their sulfonic anhydrides and their subsequent reactions with alcohols or amines to sulfonates or sulfonamides, respectively.^[^ ^49^ ^]^

Very recently, our group extended our work on electrochemical dehydration reactions to sulfonic acids (**Scheme** **16**).^[^ ^48^, ^49^ ^]^ In this work, sulfonic acids are electrochemically dehydrated to their corresponding sulfonic anhydrides. These in situ generated sulfonic anhydrides are then utilized in sulfonylation reactions: by addition of alcohols or amines, the corresponding sulfonates and sulfonamides were isolated, respectively. In total, we reported 24 substrates with isolated yields up to 67%. The scalability was also demonstrated.

**Scheme 17:**
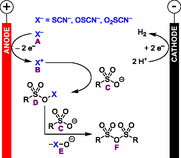
Proposed mechanism of the electrochemical dehydration of sulfonic acids to their anhydrides.^[^ ^49^ ^]^

This is the first work which applies the concept of electrochemical dehydration to sulfonic acids, as most of the previous work by us and other groups has focused on carboxylic acids. The mechanism of the electrochemical dehydration of sulfonic acids to their sulfonic anhydrides (**Scheme** **17**) is similar to our proposed mechanism for the electrosynthesis of carboxylic anhydrides from carboxylic acids. The thiocyanate‐supporting electrolyte **A** is anodically oxidized to **B** and reacts with a sulfonic acid anion **C** to an active ester **D**. Another equivalent of sulfonic acid anion **C** reacts with **D** to the corresponding sulfonic anhydride **F** under elimination of **E**. Upon addition of alcohols or amines after electrolysis (not shown in Scheme 17), the sulfonic anhydrides react in sulfonylation reactions to the corresponding sulfonates or sulfonamides, respectively.

## 8 Electrochemical Synthesis of Cyclic Sulfites from Diols Using Sulfur Dioxide Upcycling

**Scheme 18:**
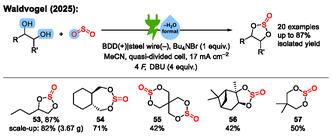
Electrochemical synthesis of cyclic sulfites from diols and SO_2_. DBU = 1,8‐diazabicyclo[5.4.0]undec‐7‐ene.^[^ ^50^ ^]^

Also recently, our group reported an electrochemical synthesis of cyclic sulfites from diols and sulfur dioxide (**Scheme** **18**).^[^ ^50^ ^]^ This work is part of the very recent progress in both the field of electrochemical dehydration reactions and the field of electrochemical multicomponent reactions using sulfur dioxide stock solutions,^[^ ^51^ ^]^ as it merges both topics in one reaction.

The work of our group on electrochemical multicomponent reactions using SO_2_ incorporation for the synthesis of highly value‐added products started with the electrosynthesis of sulfonates^[^ ^52^ ^]^ and has since then been extended to sulfamides,^[^ ^53^ ^]^ sulfonamides,^[^ ^54^ ^]^ alkenesulfonates,^[^ ^55^ ^]^ enaminyl sulfonates,^[^ ^56^ ^]^ and, most recently, cyclic sulfites.^[^ ^50^ ^]^

**Scheme 19:**
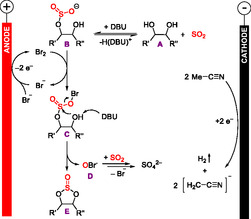
Proposed reaction mechanism of the electrochemical synthesis of cyclic sulfites from diols and SO_2_.^[^ ^50^ ^]^ DBU = 1,8‐diazabicyclo[5.4.0]undec‐7‐ene.

The proposed reaction mechanism is depicted in **Scheme** **19**. The reaction is carried out in a quasidivided cell with a steel wire as cathode. Sulfur dioxide reacts with an alcohol function of the diol **A** to a Lewis acid–base adduct (not shown here) which is deprotonated by 1,8‐diazabicyclo[5.4.0]undec‐7‐ene (DBU) to an acyclic sulfite intermediate **B**. Anodic oxidation of the bromide, which plays a dual role as supporting electrolyte and mediator,^[^ ^11^ ^]^ leads to the formation of intermediate **C** from which hypobromite **D** is eliminated as a good leaving group, while the other alcohol function of the diol attacks the sulfur atom in the acyclic sulfite intermediate to form the cyclic sulfite **E** as product. The hypobromite **D** is reduced again to bromide by sulfur dioxide. In summary, the OH group of the sulfite is converted into a good leaving group by derivatization to a hypobromite. This reaction represents both an electrochemical multicomponent reaction with sulfur dioxide and an electrochemical dehydration reaction by electrochemical (re)generation of the dehydration reagent. At the cathode, HER is observed.^[^ ^29^ ^]^ In total, we reported 20 examples with isolated yields up to 87%.^[^ ^50^ ^]^

## 9 Classification of Mechanisms

**Scheme 20:**
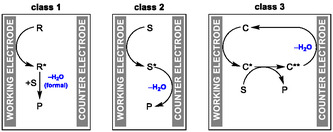
Mechanistic classification of electrochemical dehydration reactions.

Electrochemical dehydration reactions can be grouped into three different classes (**Scheme** **20**). Note that the terms working electrode and counter electrode are used, since the reaction steps of the overall dehydration could in principle proceed at the anode or the cathode. In **class 1** (Scheme 20, left), a dehydration reagent R* is generated by electrolysis from a reagent **R**, which then reacts with the substrate **S** in a subsequent nonelectrochemical dehydration step to product **P**. In the course of the reaction, one equivalent of water remains chemically bound to the dehydration reagent that was electrochemically generated. Examples developed by our group are the electrochemical dehydration of carboxylic acids or sulfonic acids to the corresponding anhydrides^[^ ^19^, ^28^, ^49^ ^]^ and the electrosynthesis of cyclic sulfites.^[^ ^50^ ^]^ The work of De Sarkar on the dehydration of primary sulfonamides with DMF derivatives to *N*‐sulfonyl amidines could also be assigned to this class.^[^ ^47^ ^]^ In **class 2** (Scheme 20, center), a domino sequence of anodic oxidation and cathodic reduction takes place (in any order), which is characterized by an overall dehydration. Here, the substrate **S** reacts at one electrode to intermediate S*, and this intermediate consecutively reacts at the opposite electrode to the product **P**. In the course of such a reaction, one equivalent of water is eliminated. This is a form of paired electrolysis.^[^ ^3^ ^]^ This sequence can be mediated, and the halogen‐mediated electrochemical dehydration of aldoximes to nitriles by Shono is a good example for such a reaction.^[^ ^38^ ^]^ An example of an unmediated case is the subsequent work of our group on this topic.^[^ ^43^, ^44^ ^]^ The electrochemical Beckmann rearrangement developed by Guan also falls under this category.^[^ ^46^ ^]^ In **class 3** (Scheme 20, right), catalytic dehydration occurs via electrochemical generation of a dehydration catalyst C* from a precatalyst **C**, which reacts with the substrate **S** to the reaction product **P** and an oxygenated species C**. The catalyst **C** is electrochemically regenerated from C** to achieve catalytic turnover. In the course of one turnover of the catalytic cycle, one equivalent of water is eliminated. An example of this type is the work by Nacsa.^[^ ^31^, ^32^, ^34^ ^]^ Finally, a clear distinction between the terms deoxygenation and dehydration must be made. The term deoxygenation is broad and it can, for example, represent a reduction reaction.^[^ ^57^ ^]^ Electrochemical deoxygenation reactions have been reviewed already.^[^ ^58^ ^]^ A dehydration in contrast is redox neutral with respect to the substrate which undergoes the dehydration reaction.

## 10 Summary and Outlook

In recent years, significant progress has been made to further develop electrochemical dehydration reactions, which are a fascinating and uprising research field within the domain of electrosynthesis. Electrochemical dehydration can either proceed via electro (re)generation of a dehydration reagent or the electrolysis results in intermediates from which water is easily eliminated without any mediator. Electrochemical dehydration reactions are especially interesting if they resemble a dehydrative coupling reaction, in which a new chemical bond is formed between two components by the removal of water, which is likely the least hazardous leaving group. Given the fundamental importance of dehydration reactions for all disciplines of chemistry, electrochemical dehydration reactions offer a more sustainable and milder alternative to hazardous dehydration reagents. Dehydration reactions are not redox reactions, because no change in oxidation state occurs in the substrate from which water is eliminated or in the dehydration reagent that is used. This could have contributed to the fact that dehydration was not viewed earlier in the context of electrolysis. The underlying idea behind this new field is to use electrolysis as a thermodynamic driving force to carry out reactions which are typically not associated with redox reactions, and this idea should serve as inspiration for other types of nonredox reactions which might be performed via electrolysis. This will lead to applications of electrosynthesis in fields where it was not considered to be the key technology before. In theory, every dehydration reaction that is currently carried out with conventional dehydration reagents could be achieved using electrochemical dehydration, which renders this new field as a promising and fascinating opportunity for many new and sustainable developments.

## Conflict of Interest

The authors declare no conflict of interest.
